# Identification of Metabolic Pathways Differentially Regulated in Somatic and Zygotic Embryos of Maritime Pine

**DOI:** 10.3389/fpls.2022.877960

**Published:** 2022-05-18

**Authors:** Concepción Ávila, María Teresa Llebrés, Vanessa Castro-Rodríguez, César Lobato-Fernández, Isabelle Reymond, Luc Harvengt, Jean-François Trontin, Francisco M. Cánovas

**Affiliations:** ^1^Grupo de Biología Molecular y Biotecnología (BIO-114), Universidad de Málaga, Málaga, Spain; ^2^BioForBois, Pôle Industrie Bois Construction, Institut Technologique FCBA, Cestas, France; ^3^BioForBois Laboratory, Pôle Industrie Bois Construction, Institut Technologique FCBA, Bordeaux, France

**Keywords:** conifers, *Pinus pinaster*, pine embryogenic lines, zygotic embryogenesis, somatic embryogenesis, transcriptome, gene regulatory networks, transcription factor

## Abstract

Embryogenesis is a complex phase of conifer development involving hundreds of genes, and a proper understanding of this process is critical not only to produce embryos with different applied purposes but also for comparative studies with angiosperms. A global view of transcriptome dynamics during pine somatic and zygotic embryogenesis is currently missing. Here, we present a genome-wide transcriptome analysis of somatic and zygotic embryos at three developmental stages to identify conserved biological processes and gene functions during late embryogenesis. Most of the differences became more significant as the developmental process progressed from early to cotyledonary stages, and a higher number of genes were differentially expressed in somatic than in zygotic embryos. Metabolic pathways substantially affected included those involved in amino acid biosynthesis and utilization, and this difference was already observable at early developmental stages. Overall, this effect was found to be independent of the line (genotype) used to produce the somatic embryos. Additionally, transcription factors differentially expressed in somatic versus zygotic embryos were analyzed. Some potential hub regulatory genes were identified that can provide clues as to what transcription factors are controlling the process and to how the observed differences between somatic and zygotic embryogenesis in conifers could be regulated.

## Introduction

Maritime pine (*Pinus pinaster* Ait.) is one of the most important forest species in southern Europe (ca. 4 million ha), particularly in the Mediterranean basin. Its significance is not only ecological but also economical, since it is a major source of wood and other forest-derived products. *P. pinaster* is widely distributed from coastal to continental areas, showing a high degree of variability and adaptability to a wide range of environmental conditions. For this reason, maritime pine is of high interest to develop efficient reproduction methods for plantation forestry purposes either through seeds or using vegetative propagation technologies to multiply true-to-type selected varieties with desirable characteristics. The control of maritime pine reproduction has also become a central issue in the context of climate change, which has led to seed shortages in this species in recent years (Boivin and Davi, 2016). Conifer somatic embryogenesis (SE) in combination with cryopreservation stands out as a powerful technology for the mass vegetative propagation of elite varieties (genotypes) with better adaptability to adverse conditions. The SE of conifers was first described in Norway spruce (Chalupa, 1985; Hackman et al., 1985) and *Larix decidua* (Nagmani and Bonga, 1985) and since then, protocols have been established for many species (Klimaszewska et al., 2016). The first report on maritime pine was published in the late 1980s (Hughes-Jarlet, 1989), and since then, the process has been extensively refined (Lelu-Walter et al., 2016; Trontin et al., 2016a). In most conifers including maritime pine, SE only allows to multiply embryos (zygote) from immature seeds. Therefore, the propagation of selected, tested varieties at field for years (typically 15 years in maritime pine) can only be obtained retrospectively from a juvenile, embryogenic cryopreserved stock established at the time of somatic embryogenesis initiation. The whole process is still difficult to achieve from other explants than seeds such as needles or buds from juvenile or adult trees (Trontin et al., 2016b). However, promising achievements have been obtained in spruce (Varis et al., 2018, and references therein).

Embryogenesis is the complex initial phase of plant life involving a network of hundreds of genes (Tzafrir et al., 2004; De Smet et al., 2010). Most knowledge has been gained from short-lived angiosperms (especially *Arabidopsis*) and little is known from perennials including gymnosperms. Yet, embryogenesis is a short but crucial phase for seed production and also for imprinting molecular patterns involved in delayed phenotypic plasticity of trees in response to environmental stress (Trontin et al., 2021). Understanding molecular aspects of both somatic (SE) and zygotic embryo (ZE) development in conifers is therefore of undoubted interest (Trontin et al., 2016c) at both fundamental and applied levels for (i) comparative studies between angiosperms and gymnosperms, (ii) understanding seed development and (iii) to optimize SE production for conservation, rescue, breeding and deployment issues of selected or natural genetic resources.

A critical process during the maturation of maritime pine embryos (from late embryogenesis to fully mature embryos) is the synthesis and deposition of storage proteins that are extremely rich in the amino acid arginine (Allona et al., 1994; Llebrés et al., 2018a,b). Somatic embryos have been reported to accumulate fewer storage proteins than fully matured zygotic embryos with an imbalance in arginine metabolism (Morel et al., 2014; Llebrés et al., 2018b).

Another pathway of paramount importance for plant N economy is the biosynthesis of aromatic amino acids (Lynch and Dudareva, 2020). Phenylalanine and tyrosine are the precursors of a variety of essential secondary metabolites for plant development and response to environmental stimuli (Pascual et al., 2016; El-Azaz et al., 2021). The enzymes arogenate/prephenate dehydratases (ADT/PDT) catalyze the rate-limiting step in phenylalanine biosynthesis, and in maritime pine, are encoded by a gene family of at least nine members (El-Azaz et al., 2016, 2021). Tyrosine is synthesized by arogenate dehydrogenase (ADH) using an intermediary of the arogenate pathway (Schenck and Maeda, 2018; Lynch and Dudareva, 2020).

Transcriptomic approaches in conifers have been very useful in understanding both basic functions in tree biology and global responses to environmental changes. In maritime pine, the availability of genetic and genomic resources has largely allowed their use in functional genomics approaches (Cañas et al., 2019).

In this work, RNA-seq was used to further improve a previous version of the maritime pine transcriptome (Canales et al., 2014; Cañas et al., 2017) and, additionally to understand and compare the embryo maturation during somatic and zygotic late embryogenesis at the transcriptional level. The aim was to specifically compare somatic and zygotic embryos to discover differentially expressed genes and metabolic pathways functioning in a different manner during late somatic embryogenesis. Three key developmental stages were analyzed for both SE (from early to cotyledonary stages) and ZE (from pre-cotyledonary to cotyledonary embryos).

Significant differences were found in the expression profiles of SE. Special emphasis was placed on the identification of key transcription factors (TFs) involved in the regulation of the process and amino acid biosynthesis pathways vitally important for embryo development. Overall, the results provide new insights into conifer embryogenesis, with potential value to better understand seed development in the context of global warming and refine the vegetative propagation of maritime pine via SE toward multi-varietal forestry (Lelu-Walter et al., 2016; Trontin et al., 2016a).

## Materials and Methods

### Plant Material

The *P. pinaster* embryogenic cell line PN519 (controlled cross G0.4304*G0.4301) selected for this work was initiated in 1999 (ref. 99PP1) at FCBA (France) and has been extensively characterized during the past 15 years (Trontin et al., 2007, 2016a; Lelu-Walter et al., 2016; Llebrés et al., 2018b). Proliferation was performed on modified Litvay medium (mLV) with low PGRs as defined by Klimaszewska et al. (2001). For the maturation step of somatic embryos, the mLV medium was supplemented with higher content of sucrose (68 g L^–1^) and, gellan gum (Phytagel, 9 g L^–1^), and abscisic acid (ABA, 80 μM) was used as the only plant growth regulator. Proliferation and maturation were conducted at 24°C in darkness inside a culture chamber. Samples were collected at three different stages of maturation: early-stage translucent SE (ES1, after 4–6 weeks), pre-cotyledonary opaque SE (ES2, after 6–10 weeks) and cotyledonary SE (ES3, after 12–14 weeks). In addition to PN519, three embryogenic cell lines ABN20008, ABN200010, and ABN20004 initiated in 2020 at FCBA (ref. 20PP7, 20PP8, and 20PP10) from open-pollinated mother tree G0.0123 were also analyzed. Samples were collected at FCBA for two stages of maturation: pre-cotyledonary opaque SE (ES2) and cotyledonary SE (ES3), frozen in liquid nitrogen and stored at −80°C until use. For simplification we will refer to the PN519, ABN20008, ABN200010, and ABN20004 lines as follows throughout text: PN519, PP7, PP8, and PP10, respectively.

Reference zygotic embryos (ZE) were excised from seeds collected from a single maritime pine (*Pinus pinaster* Ait.) seed orchard (PP-VG-014, Picard, Saint-Laurent-Médoc, France) from July to November 2015. Embryos were sampled at different developmental stages according to de Vega-Bartol et al. (2013): pre-cotyledonary ZE (PC, early to mid-July), early cotyledonary ZE (EC, mid to late-July) and cotyledonary immature ZE (C, from early August to early September). The ZE samples were frozen in liquid nitrogen and stored at −80°C until use.

### RNA Extraction and Sequencing

Extraction of total RNA was performed as described by Canales et al. (2012) and initially quantified using a NanoDrop© ND-1000 spectrophotometer. Biological samples representing PC, EC, and C stages of ZE development were harvested. In addition, equivalent stages (ES1, ES2, and ES3) of the maturation phase of PN519 line were selected also for RNA isolation. Three technical replicas of three independent biological samples were used in all cases. The RNA samples were tested for quantity and integrity using a 2100 Bioanalyzer (Agilent, Santa Clara, CA, United States). Only RNA with RNA integrity number (RIN) 7 or higher was used for subsequent processing. Synthesis of cDNA, construction of cDNA libraries and HiSeq was performed by Novogene (Hong Kong) using a NovaSeq 6000 sequencer according to manufacturer instructions for paired-end reads of 150 bp length (Illumina, San Diego, CA United States). RNA from samples corresponding to lines PP07, PP08, and PP10 was extracted as previously described.

### Data Pre-processing and Transcriptome Assembly

Once the raw data was obtained, a reading cleaning process was carried out to remove contaminants, low quality readings and adapters not previously removed using SeqTrimBB^1^. A quality filter Q > 20 was applied as previously described (Ortigosa et al., 2021). Maritime pine reference transcriptome (Canales et al., 2014) was implemented with sequencing data produced during this study (Supplementary Table 1). Reads were assembled using Trinity 2.11 (Haas et al., 2013) as described in Ortigosa et al. (2021). Redundancy was reduced using CD-HIT-EST (Li and Godzik, 2006). This preliminary assembly was the input of the Mira assembler software (Chevreux et al., 2004). The maritime pine transcriptome (Canales et al., 2014; Cañas et al., 2017) was used as the reference for the read mapping that was performed with BWA using the MEM option (Li and Durbin, 2009). Expression level was obtained by reading counting using the phyton script *sam2counts*^2^. Differentially expressed (DE) transcripts were identified using the edgeR package for R, and then normalized by cpm and filtered with 2 cpm in at least 6 samples (Robinson et al., 2010).

Samples were grouped by SE or ZE stage. After differential expression analysis only transcripts with FDR < 0.05 and a Fold Change >1 were considered as differentially expressed. These RNA-seq data have been deposited in the NCBI’s Gene Expression Omnibus (Edgar et al., 2002) and are accessible through GEO Series under the accession number GSE194039^3^. Additionally, RNA seq and network results are accessible through a database in HTML format that can be installable with R packages and downloaded from GitHub^4^.

Subsequently, functional enrichments were made using the annotations of “Gene Ontology (GO)” and “KEGG Orthology (KO)” using AgriGO computer tools (Tian et al., 2017), as it is the most suitable for this type of processes in plants, and KEGG Mapper (Kanehisa and Sato, 2020), respectively.

### Real-Time Quantitative PCR

Synthesis of cDNA was performed using total RNA (500 ng) and with 5X iScript™ cDNA Synthesis Kit (Bio-Rad) in a volume reaction of 10 μL following manufacturer’s recommendation. Real-time PCR was carried out on a CFX384 thermal cycler (Bio-Rad) under the following conditions: 1 cycle of denaturation at 95°C for 2 min, 40 cycles of denaturation at 95°C for 1 s, hybridization at 60°C for 5 s and finally 1 cycle of 30 s at 95°C, 5 s at 65°C, 0.5 s at 95°C for the generation of the dissociation curve that confirmed the specific amplification of each reaction. Each reaction proceeded by triplicate in a total volume of 10 μL, 5 μL of SsoFast™ EvaGreen^®^ Supermix (Bio-Rad), 2 μL cDNA (5 ng μL^–1^) and 0.5 μL of 10 μM of a specific primer. *Actin-7* (18113) was used as a reference gene. Sequences of specific primers are listed in Supplementary Table 2. Relative expression profiles for each gene were obtained employing the R package (Ritz and Spiess, 2008) and normalized to the reference gene. This procedure was used first to validate level of expression inferred from the RNA seq data (Supplementary Figure 1) and afterward for expression analysis of candidate genes in the four embryonic lines used in this study at ES2 and ES3 stages compared to their counterparts zygotic embryos.

### Construction of Co-expression Networks and Mining of Hub Genes

Using the normalized data from the RNA-seq reads, the co-expression networks were established. In this way we can obtain the grouped genes thanks to their joint expression profiles using a statistical correlation test. WGCNA R (R Core Team, 2021) package was necessary for the co-expression network construction (Langfelder and Horvath, 2008). The function pickSoftThreshold was used to choose an appropriate soft-thresholding power based on a scale-free topology criterion. The weighted adjacency matrix was constructed using the soft-thresholding power. The relationships of the elements within these groups allow us to know those genes involved in the regulation of the candidate genes obtained in the previous analysis of RNA-seq and/or in the biological pathways of interest. These “hub genes” are those that maintain a greater number of significant interactions with the rest of the elements within the co-expression groups. To determine these “hub genes,” the topological adjacency matrix generated by WGCNA was used to determine those genes that have high connectivity, that is the 10% of the genes with more connections within each module and that have a high affiliation value per module (Yang et al., 2019). The identification of regulatory elements that take part in biological pathways of interest is intended.

The relationship between one gene and all other ones in the analysis was incorporated, and the adjacency matrix was transformed into the topological matrix (TOM) (Yip and Horvath, 2007). After hierarchical clustering, highly interconnected genes were assigned to the same module (Ravasz et al., 2002). Hub genes were extracted selecting the 10% of the genes with more connectivity of each module and gene Module Membership > 0.80 (Wang et al., 2021).

## Results

### Experimental Design and Gene Expression Patterns During Pine Development

**FIGURE 1:**
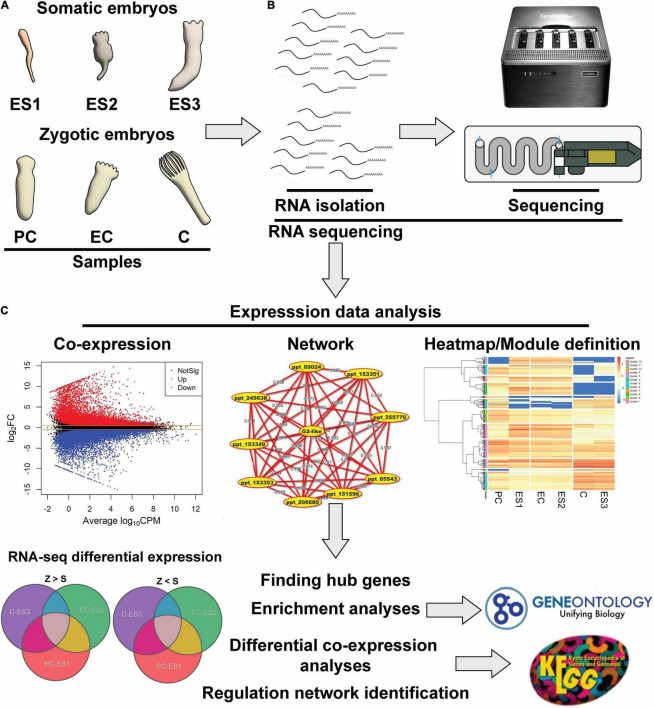
Experimental design. **(A)** Schematic representation of comparative developing stages of somatic: early-stage translucent (ES1), pre-cotyledonary opaque (ES2), and cotyledonary (ES3) and zygotic embryos: pre-cotyledonary (PC), early cotyledonary (EC) and cotyledonary (C). **(B)** Scheme of RNA extracted from samples and subsequent NGS-sequencing. **(C)** Differential expression analysis using bioinformatics tools.

To perform a global study of gene expression in pine embryos (Figure 1), two sets of samples were analyzed representing comparative developmental stages in somatic early-stage translucent (ES1), precotyledonary opaque (ES2) and cotyledonary (ES3) embryos, and zygotic precotyledonary (PC), early cotyledonary (EC) and cotyledonary (C) embryos (Llebrés et al., 2018b). Samples were used to extract high-quality RNA for sequencing using the Illumina platform (Figures 1A,B). Expression information from samples harvested during the time course of embryo development corresponded to the total genes expressed either in zygotic or somatic pine embryos in all developmental stages considered. These transcriptomic data were further used for differential expression analysis, gene-network construction and heat-map/module definition (Figure 1C).

**FIGURE 2:**
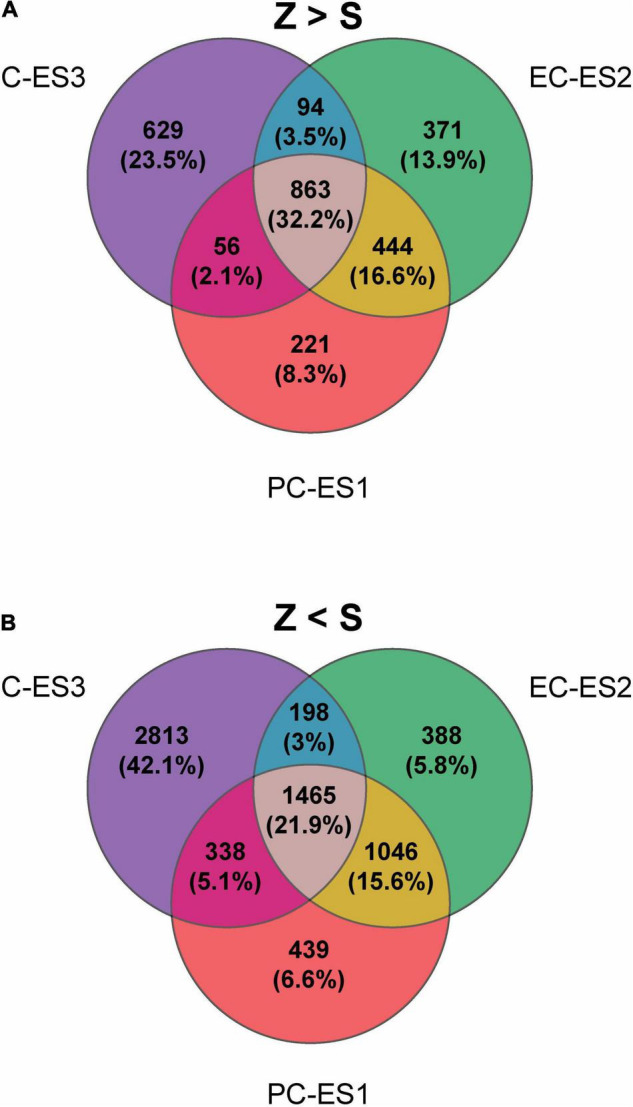
Global differential expression analyses. Venn diagrams of differentially expressed genes in zygotic (Z) versus somatic (S) embryos in three consecutive sets of similar developmental stages during late embryogenesis: PC and ES1, EC and ES2, C and ES3, respectively. The number of genes showing a fold-change >2 between consecutive stages of development is shown. **(A)** Genes overrepresented in zygotic (Z) compared the somatic (S) stages are considered. **(B)** Genes underrepresented in the zygotic are considered.

The global differential expression analysis is presented in Figure 2. A Venn diagram depicting the number and percentage of overrepresented genes in zygotic versus somatic embryos is presented in Figure 2A. The number of genes overrepresented in the zygotic embryos is similar in all different developmental stages (PC, 1584; EC, 1772; C, 1642). In contrast, the number of overexpressed genes in somatic embryos was greater than that observed in zygotic development (6,687 in total, Figure 2B). There are more genes overrepresented in stage ES3 (4,814) compared to ES1 (3,288) and ES2 (3,097). These findings indicate that several pathways were upregulated in SEs from early to late developmental stage compared to their presumed zygotic counterparts. Transcriptomic data were validated by qPCR analysis of selected genes (Supplementary Figure 1).

### Co-expression Analysis of Differentially Expressed Genes

**FIGURE 3:**
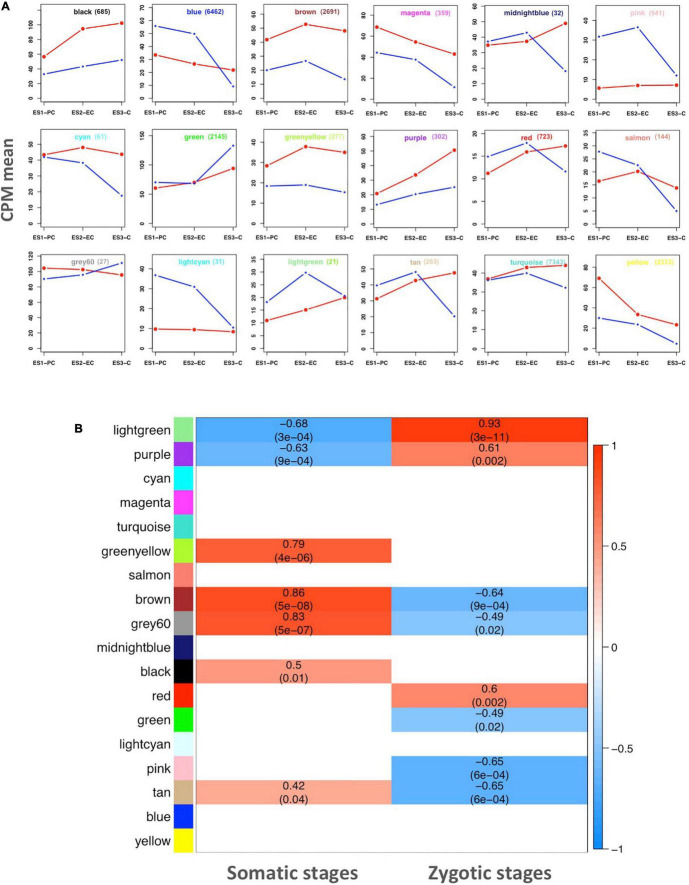
Gene co-expression network analysis. **(A)** Mean expression profile of genes in each correlation module representing correlative developing stages (CPM, counts per million mapped reads). The number of genes included in each module is shown by its name. Zygotic developmental stages: PC, EC, and C represented in blue. Somatic developmental stages: ES1, ES2, and ES3 are represented in red. Data are means of three values from the RNA seq reactions. **(B)** Heat map showing the relationships between the modules and embryo developmental stages. The correlation, positive (light to dark red) or negative (light to dark blue) with either somatic or zygotic embryos data globally represented is indicated.

To identify major trends in both sets of genes, a cluster analysis based on co-expression during embryo development was performed. Hierarchical clustering was used to group the DEGs into a minimal set of clusters (modules) reporting conserved expression profiles within each cluster and distinct profiles among clusters. The hierarchical clustering grouped the set of genes into eighteen modules, each of which was identified with a color: black, blue, brown, magenta, midnight blue, pink, cyan, green, greenyellow, purple, red, salmon, gray60, lightcyan, lightgreen, tan, turquoise, yellow, and in addition a false gray module representing unassigned genes (eight genes) that does not represent a real module (Figure 3). The genes assigned to each module are listed in Supplementary File 1. The number of genes integrating each module and the mean expression levels of genes in each module are shown in Figure 3A. Modules with the greatest number of genes were turquoise (7,343) and blue (6,462). The smallest modules include midnight blue (32), lightcyan (31), gray60 (27), and lightgreen (21).

Eight modules displayed genes overrepresented in somatic versus zygotic embryos, regardless of the developmental stage: black, brown, magenta, cyan, greenyellow, purple, turquoise, and yellow. Three modules grouped genes underrepresented in the somatic embryos: pink, light cyan and lightgreen. The rest of the modules represent groups of genes whose expression changed during the development of somatic embryos from being over- or underrepresented in the early stages of development (ES1) to being under- or overrepresented in the later stages (ES2 and ES3).

The relationship of the modules either with somatic or zygotic embryos was performed using a Pearson correlation and examined generating a heatmap shown in Figure 3B. The lightgreen module was strongly correlated with both somatic (negatively) and zygotic stages (positively). However, this module has only 21 genes with no significant functional, defined categories. Another module, purple (302 genes), similarly correlated with both somatic (negatively) and zygotic (positively) stages. Conversely, three modules were positively correlated with somatic stages and negatively with zygotic stages: brown (2,691 genes), gray60 (27), and tan (263). These modules include important genes involved in development, cellular biogenesis and regulation. There are two modules that only showed positive correlation with somatic stages: greenyellow (277 genes) and black (685) involved in metabolic processes and regulation. Similarly, two modules showed positive correlation but only with zygotic stages: red (723 genes) and the false module gray (8). At last, two modules also correlated with zygotic stages but negatively: green (2,145 genes) and pink (541). These modules representing a specific correlation may include genes that are specifically involved in either somatic or zygotic developmental stages.

To determine functions associated with the modules, enrichment analysis was performed using the AgriGO platform (Tian et al., 2017). Distribution of functional categories corresponding to each co-expression module is included in Supplementary File 2. It is worth highlighting the blue module made up of 6,462 genes in which there is a broad representation of genes involved in regulation of biological processes: transcriptional regulation (518), regulation of gene expression (628) and regulation of metabolic processes such as the metabolic regulation of nitrogenous compounds (574). Similarly, the large module turquoise (7,343 genes), has a considerable representation of genes involved in development (326), morphogenesis, division and cell proliferation, and regulation of metabolic processes (732) such as transport, nutrient reservoir and catalytic activity, and stimulus response (603). Modules containing approximately 100 or fewer genes did not have characteristic functional enrichments except for the salmon module integrated by 144 genes, which presented enrichment in 49 genes involved in oxidation/reduction. To further understand functions at the molecular level, a KEGG mapping was performed for a global metabolic overview. Several metabolic pathways were highlighted being more represented in somatic embryos such as those related to N metabolism (Supplementary Figure 2) and biosynthesis of aromatic amino acids and phenylpropanoids (Supplementary Figure 3).

### Search for Hub Genes During Maritime Pine Embryo Development

**FIGURE 4:**
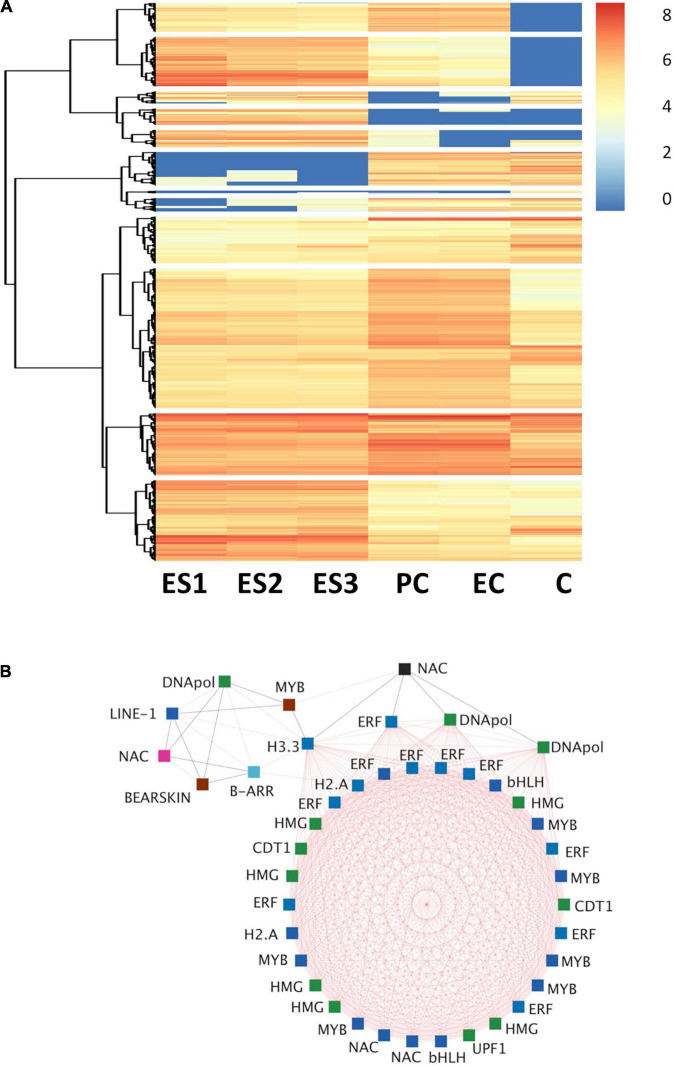
Identification of hub genes and transcription factors. **(A)** Hierarchical clustering and heat map of hub genes differentially expressed in somatic: ES1, ES2, and ES3 and zygotic: PC, EC, and C embryos. The heat map represents a total number of 1657 hub genes with connectivity >10% (see section “Materials and Methods”). The hierarchical clustering was performed using Ward’s minimum variance method. **(B)** Co-expression network interactions of TF hub genes differentially expressed during pine embryo development. TFs included are: NAC domain protein (NAC), Myb-related protein (MYB), ethylene-responsive TF (ERF), protein BEARSKIN, high mobility group B protein (HMG), histone H3.3, histone H2A, chromatin licensing and DNA replication factor 1 (CDT1), DNA polymerase (DNApol), regulator of nonsense transcripts 1-like protein (UPF1), bHLH, LINE-1 reverse transcriptase isogeny (LINE-1) and type-B response regulator (B-ARR). Cytoscape version 3.9.1 platform was used to visualize the network (Shannon et al., 2003). Square symbols mean TF, blue edges indicate negative co-expression associations and red edges indicate positive co-expression associations. Colors inside each symbol agree with the co-expression module where the gene is included (see Figure 3).

Next, we searched for genes with a high level of connectivity in each module. As described in section “Materials and Methods” the 10% of the transcripts with more connections were considered hub genes. Using this criterion, a total of 1,657 hub genes were listed across modules and annotated in Supplementary Table 3, and further characterized (Figure 4). Establishing a hierarchical cluster of hub genes based on the whole dataset, we obtained a heatmap showing the relationship of these highly connected genes with the corresponding embryo developmental stage. As shown in Figure 4A, a greater generalized expression of the hub genes was observed at early stages of the development in both zygotic and somatic embryos.

To gain insights into the regulation of maritime pine embryos special attention was given to the identification of TFs that were at the same time hub genes (Supplementary Table 4). The presence of B-ARR and ERF protein families, whose members have been described as participating in the regulation of plant development, was particularly noteworthy. A total of 63 hub genes were classified as TFs, and their distribution in the different co-expression modules was uneven. The module with the highest number of hub genes that are at the same time TFs was the blue module with 34 regulatory genes belonging to the NAC, ERF, Myb, and b-Zip families, although the most abundant were included in the ERF family with 10, and the MYB family with 6 representatives. The other modules with the highest representation of hub regulatory genes were the green module, with 13 genes mainly belonging to the HMG group, and the turquoise module, with eight members, two of which were B-ARR. There were some modules without representation of hub regulatory genes, mainly including those that were integrated by a small number of genes: cyan, greenyellow, gray60, lightcyan, lightgreen, midnight blue, pink, purple, red, salmon, or tan.

**FIGURE 5:**
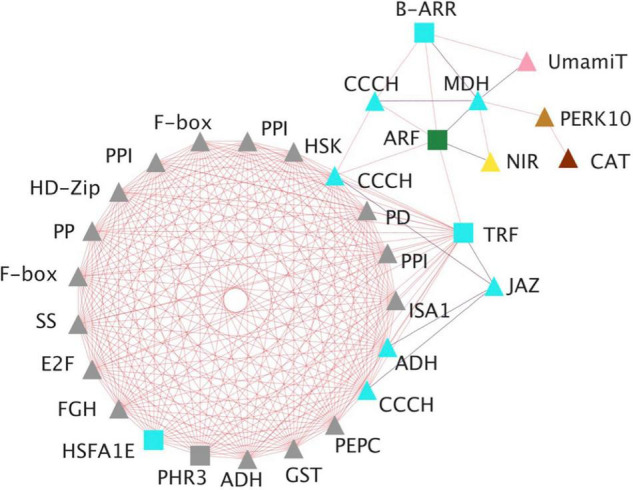
Graphical view of the *B-ARR* gene co-expression network. Genes included are those encoding: E2F, E2F-like protein; PHR3, protein phosphate starvation response 3; PERK10, proline-rich receptor-like protein kinase; PPI, peptidyl-prolyl *cis-trans* isomerase; GST, glutathione S-transferase; F-box, F-box/kelch-repeat protein; JAZ, jasmonate ZIM-domain protein; PEPC, phosphoenolpyruvate carboxylase; PP, serine/threonine-protein phosphatase; PD, prephenate dehydrogenase; ADH, alcohol dehydrogenase; HSK, homoserine kinase; FGH, S-formylglutathione hydrolase; MDH, malate dehydrogenase; CCCH, zinc finger CCCH domain; HD-Zip, homeobox-leucine zipper-like protein; RABA1f, ras-related protein; ISA1, isoamylase 1; NIR, ferredoxin–nitrite reductase; HSFA1E, heat stress TF A-1e; GST, glutathione S-transferase; ARF, auxin response factor; CAT, cationic amino acid transporter, usually multiple amino acids move in and out transporter (UMAMIT), starch synthase (SS) and telomeric repeat binding protein (TRF). Cytoscape version 3.9.1 platform was used to visualize the network (Shannon et al., 2003). Square symbols mean TF, triangles mean no-TF, blue edges indicate negative associations and red edges indicate positive associations. Color inside each node means the co-expression module where the gene is included. Gray symbols represent genes that in the B-ARR interaction network are only expressed in the cotyledonary stage (C) of the zygotic embryos.

A gene co-expression network analysis was performed for relevant hub TFs exhibiting the highest number of connections to construct a network diagram (Figure 4B). The aim was to provide a greater understanding of the cellular and metabolic functions related to the selected TFs. The co-expression network include TFs mainly of the NAC, MYB, ERF, HMG, and bHLH families and their expression increased during the development of somatic embryos. Among them, a member of B-ARR was identified acting as a main regulator of the network. Figure 5 shows the co-expression network of *B-ARR* from the turquoise module. This TF is related to other regulatory and structural genes involved in nutrition: (i) nitrogen, nitrite reductase, cysteine synthase, and transporters such as UMAMIT and CAT; (ii) carbon, starch synthase, phosphoglycerate kinase, phosphoenolpyruvate carboxylase, ribulose bisphosphate carboxylase small subunit, sedoheptulose-1,7-bisphosphatase; and (iii) sulfur and phosphate, sulfate transporter 3.1, and protein phosphate starvation 3 as well as other TFs such as CCCH zinc fingers, JAZ and HD-Zip members. As nutrition is essential for embryo development the functional characterization of *B-ARR* deserves special attention.

### Validation of Differentially Regulated Pathways in Several Lines of Somatic Embryos

As transcriptome and co-expression network analyses of zygotic and somatic embryos were performed in a single embryogenic line (PN519), subsequent experiments were performed to elucidate whether the observed differences could be related to the SE protocol or rather to the characteristics of the embryogenic line itself. The transcript levels of regulatory and structural genes were assessed in PN519 as well as in 3 additional embryogenic lines (PP7, PP8, and PP10). PN519 is routinely used at different laboratories since 1999 and typically exhibits high embryogenic ability (mean of 94 SE g^–1^ fresh mass embryogenic tissue, Lelu-Walter et al., 2016; Trontin et al., 2016a). In contrast, PP7, PP8, and PP10 are 3 lines initiated more recently (2020) and showing quite low embryogenic ability (PP7: 16 SE g^–1^; PP8: 26 SE g^–1^; PP10: 19 SE g^–1^). Gene expression was studied at stages ES2 (precotyledonary) and ES3 (cotyledonary) of SE and stages EC (early cotyledonary) and C (cotyledonary) of ZE.

**FIGURE 6:**
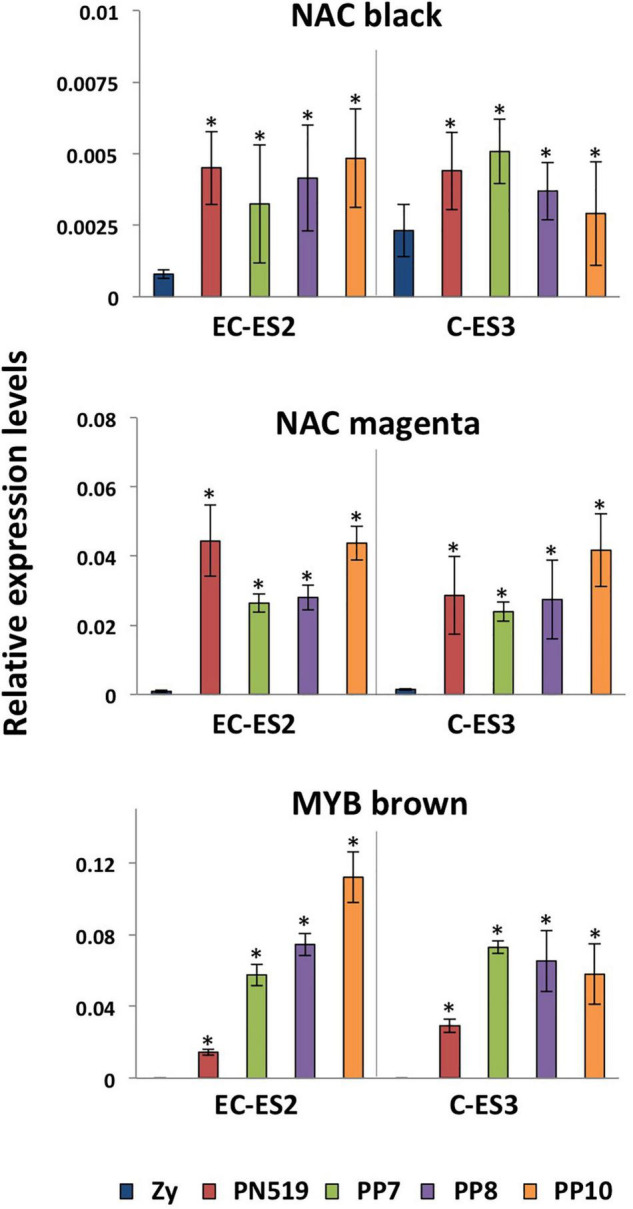
Expression patterns of regulatory hub genes. Quantitative analysis of transcript levels for genes that are differentially expressed in zygotic embryos (Zy) at early cotyledonary (EC) and cotyledonary stages (C) versus four somatic lines, PN519, PP7, PP8, and PP10, at pre-cotyledonary opaque (ES2) and cotyledonary (ES3) stages. Genes included in the analysis were those encoding NAC black, NAC magenta, and MYB brown in reference to the co-expression module where the TF is included. Each value is the mean ± standard deviation of three biological replicates. Asterisks indicate significant differences between zygotic and somatic samples calculated using Student’s *t*-test (*P* < 0.05). The expression level for all genes was normalized using Actin-7 as a reference.

Figure 6 shows the expression levels of several hub regulatory genes previously identified by gene network analysis. No major differences were observed in the transcript levels of two NAC genes among the SE lines examined. In contrast, a MYB from the brown module depicted increased levels of expression in the three SE lines that exhibits a lower embryogenic capacity.

**FIGURE 7:**
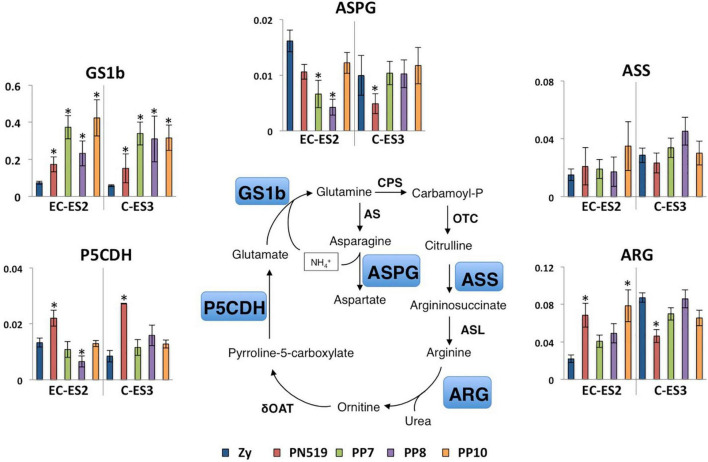
Differential expression of genes involved in arginine metabolism. Quantitative analysis of transcript levels for genes that are differentially expressed in zygotic embryos (Zy) at early cotyledonary (EC) and cotyledonary stages (C) versus four somatic lines, PN519, PP7, PP8, and PP10, at pre-cotyledonary opaque (ES2) and cotyledonary (ES3) stages. Genes included in the analysis were those encoding glutamine synthetase (GS1b), asparaginase (ASPG), argininosuccinate synthase (ASS), arginase (ARG) and pyrroline-5-carboxylate dehydrogenase (P5CDH). Each value is the mean ± standard deviation of three biological replicates. Asterisks indicate significant differences between zygotic and somatic samples calculated using Student’s *t*-test (*P* < 0.01). The expression level for all genes was normalized to that of Actin-7 as a reference gene.

Considering that N is an important nutrient involved in embryo development, the amino acid metabolism was also studied. Gene expression related to arginine metabolism is shown in Figure 7. The relative abundance of transcripts for glutamine synthetase (GS1b) in the four embryogenic lines was higher than that in zygotic embryos at either the EC-ES2 or C-ES3 stage. In contrast, the expression levels of *Asparaginase* (*ASPG*) were lower in the four lines at the EC-ES2 stage, although similar expression levels were found at the C-ES3 stage, with the exception of the PN519 line, which still exhibited lower transcript abundance than the zygotic line. *Argininosuccinate synthetase* (*ASS*) transcripts displayed a slight increase in the embryogenic lines compared to those observed in the zygotic samples for both the EC-ES2 and C-ES3 stages. *Arginase* (*ARG*) expression showed a clear induction at the stage EC-ES2 for all embryogenic lines (as *GS1b*), although in C-ES3, no major differences were observed between lines and zygotic embryos, except for PN519 showing a different pattern of reduced expression. PN519 was the unique line showing increased levels of transcripts encoding delta-1-pyrroline-5-carboxylate dehydrogenase (P5CDH) in zygotics at both stages examined.

**FIGURE 8:**
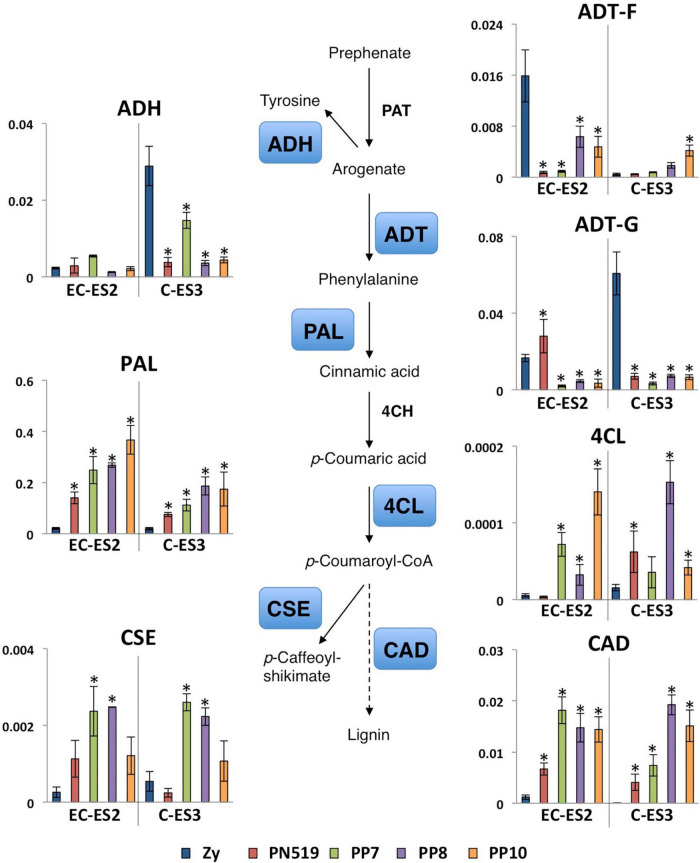
Differential expression of genes involved in phenylalanine biosynthesis and utilization. Quantitative analysis of transcript levels for genes that are differentially expressed in zygotic embryos (Zy) at early cotyledonary (EC) and cotyledonary stages (C) versus four somatic lines, PN519, PP7, PP8, and PP10, at pre-cotyledonary opaque (ES2) and cotyledonary (ES3) stages. Genes included in the analysis were those encoding arogenate dehydratase (ADT-F and ADT-G isoforms) ADH, arogenate dehydrogenase; PAL, phenylalanine ammonia-lyase; 4CL, 4-coumaroyl-CoA ligase; CSE, caffeoyl shikimate esterase; and CAD, cinnamyl alcohol dehydrogenase. Each value is the mean ± standard deviation of three biological replicates. Asterisks indicate significant differences between zygotic and somatic samples calculated using Student’s *t*-test (*P* < 0.01). The expression level for all genes was normalized to that of Actin-7 as a reference gene.

The differential expression of genes involved in the biosynthesis and metabolic fate of phenylalanine and tyrosine is shown in Figure 8. No changes in the expression of *ADH*, a gene involved in tyrosine biosynthesis, were apparent at stages EC-ES2. However, decreased levels of *ADH* transcripts in the four embryogenic lines were recorded when compared to those in zygotic embryos later in development (C-ES3). The expression levels of two members of the *ADT* gene family were also assessed: ADT-F, a typical ADT from conifers with unknown function, and ADT-G, an arogenate dehydratase also exhibiting prephenate dehydratase (PDT) activity (El-Azaz et al., 2016). *ADT-F* and *ADT-G* expression levels were lower at both stages in the embryogenic lines except for line PN519, which exhibited higher expression of *ADT-G* at EC-ES2. Phenylalanine is the precursor of many secondary metabolites and structural components in vascular plants, such as lignin. Regarding metabolic utilization of phenylalanine for the biosynthesis of phenylpropanoids all genes examined exhibited enhanced expression levels in the four embryogenic lines (Figure 8).

## Discussion

A transcriptomic general survey previously performed in maritime pine zygotic embryos revealed the high representation of transcripts involved in carbohydrate metabolism, monosaccharide transport and the possible role of epigenetic regulation (de Vega-Bartol et al., 2013; Rodrigues et al., 2018). The aim of this work was to have an overview of the transcriptome dynamics in both systems, to identify pathways/genes that are differentially represented, and which regulators of gene expression could be identified with an emphasis on regulatory hub genes, i.e., those with the highest interactions within co-expression groups. The final goal was to pave the way for subsequent comparative studies of zygotic and somatic embryogenesis in plants with a biotechnological, practical dimension addressed to improve the quality and ultimately the vigor of SE plants. Somatic embryogenesis in maritime pine has been significantly refined but one major problem remaining is low initial vigor of somatic seedlings at field compared to standard, reference seedlings (Trontin et al., 2016a). Although the somatic seedlings regain normal growth after 2–3 seasons, the initial time lag is a major limitation for the practical implementation of the technology by foresters, particularly considering the competition with weeds which is very strong the first year after planting.

### What Have We Learned From the Transcriptome Differential Analysis?

Transcriptome analyses have shown that the number of genes differentially expressed during the maturation of zygotic embryos does not change substantially when considering the precotyledonary, early cotyledonary or cotyledonary stages. Approximately 1600 genes in each case were overrepresented during zygotic embryo development, and 50% of them were common in all three stages, showing functions that were found to be underrepresented in the development of somatic embryos (Figure 2). Conserved functions predominantly represented in zygotic over somatic embryos were cellular metabolic processes involving macromolecules, nucleotides and nucleic acids, which are necessary to assist the growth of new plants (Supplementary File 3). These findings agree with those of a previous study of transcriptome dynamics in *Pinus sylvestris* zygotic embryos (Merino et al., 2016). The number of genes exclusively overrepresented in each zygotic phase increased from the precotyledonary stage to the cotyledonary stage as expected for a process of increasing complexity as the embryo develops. The number of DEGs overexpressed in common between stages was highest for the PC and EC stages suggesting that these 2 stages are less differentiated compared to the C stage in accordance with de Vega-Bartol et al. (2013). In contrast, the number of genes overrepresented in the somatic embryos was higher than in zygotic embryos, increasing from 3,000 genes in stages ES1 (early stage) and ES2 (pre-cotyledonary) up to 4,800 in ES3 (cotyledonary). It would be interesting to see how these differences translate at the functional proteomic level. Functional categories for common genes in somatic stages were those related to developmental and metabolic processes and regulation of metabolic processes (Supplementary File 4). Remarkably, a high number of genes involved in regulation were differentially represented in somatic embryos. The gene co-expression modules overrepresenting functions in somatic embryos were consistent with the global transcriptomic analyses previously performed. In fact, eight modules contain genes with a higher expression level in somatic embryos at all developmental stages examined (Figure 3). These results clearly indicate that changes in the transcriptome were much more pronounced in somatic than in zygotic embryos. Some metabolic pathways were found more represented in somatic embryos such as nitrogen metabolism, biosynthesis of aromatic amino acids and phenylpropanoids. There was also prevalence of genes involved in oxidation-reduction processes. Nitrogen-related metabolic variations are key issues during conifer somatic embryogenesis up to the germination phase (Pérez-Rodríguez et al., 2006; Dowlatabadi et al., 2009; Businge et al., 2013; Llebrés et al., 2018b). In addition, pathways related to secondary metabolisms such as phenylpropanoids or oxidation-reduction have been involved in stress resistance processes (defense functions) associated with embryo development such as maintenance of efficient cellular homeostasis [reviewed in Trontin et al. (2016c)]. Taken together, the above findings suggest that during late embryogenesis there is a readjustment in transcriptome dynamics allowing compliance with the developmental program that is well established during embryogenesis. To gain insights into how gene expression is reprogrammed during somatic embryo development a systems biology approach was followed to identify relevant regulatory genes involved in the observed changes.

### Identification of Hub Genes and Molecular Signaling in Maritime Pine Embryos

Information processing is essential to ensure that an efficient response can be translated into development. In regulation, signaling linear cascades are combined in multidimensional networks to develop greater specificity and allow successful integration of signals (Milo et al., 2002). The data shown in Figure 4 indicate that most identified hub genes were highly expressed at early stages of embryo development, either somatic or zygotic. These results suggest that molecular and functional interactions are essential during the initial steps of embryo development since there are many processes that must start to guarantee that the final cotyledonary, mature embryo is fully functional. Furthermore, the transcriptional analyses revealed that many TFs were differentially expressed during somatic and zygotic embryogenesis and allowed to identify those that were hub genes themselves. Although their functions must be validated, it is interesting to highlight the identification of B-ARR as one of the TFs with the highest connectivity among the hub regulatory genes. *B-ARR* genes are members of the GARP family of TFs initially identified in *Arabidopsis* as transcriptional effectors downstream of cytokinin network signaling (Kieber and Schaller, 2014; Xie et al., 2018). Cytokinin is a phytohormone that is involved in almost every aspect of plant growth and development. It was first characterized by its ability to promote cell division (Skoog and Miller, 1957), and since then, its roles in many cellular processes have been reported (Kieber and Schaller, 2014). In conifers, there is now some discussed evidence that cytokinins are involved in maintenance of the embryogenic state (Gautier et al., 2019), embryo development (Li et al., 2017) as well as transduction of environmental signals resulting in plant phenotypic plasticity (Castander-Olarieta et al., 2021; Trontin et al., 2021). The GARP family includes regulatory genes involved in the control of plant responses to nutrients and it has been proposed to also include nutrient sensors (Safi et al., 2017). The network generated by this hub gene includes TFs belonging to other families, which suggests a multitude of transcriptional controls over other pathways. It is noteworthy that the regulation of transport and mobilization of amino acids during embryogenesis (Llebrés et al., 2018b) and seed development (Besnard et al., 2018) are important N bottlenecks in pine. In this sense, the identification of transporters such as UMAMIT in the *B-ARR* gene network provides new insights into the availability, transport and use of N at early stages of pine embryo development.

### Regulatory and Structural Genes Were Differentially Regulated in Several Somatic Embryogenic Lines

While the above results provide new insights into how maritime pine SE is regulated, they have the potential limitation of being acquired using a single embryogenic line with robust ability to produce cotyledonary embryos (PN519). To confirm the data derived from the system genetics approach, the transcript levels of candidate genes were validated by quantitative analyses in distinct embryogenic lines with lower embryogenic ability.

Differences were observed in the relative expression of regulatory genes, that allowed the identification of a Myb TF likely associated to the distinct embryogenic capacity of the lines (Figure 6). These findings points to that the functional dissection of the hub gene networks can provide valuable knowledge for improving the maturation of somatic embryos.

As the availability of organic N is strongly connected with the growth and development of forest trees (Castro-Rodriguez et al., 2016; Cánovas et al., 2018), metabolic pathways related to amino acid metabolism were also selected for validation analysis. Moreover, several key enzymes of amino acid metabolism, such as N-acetyl glutamate kinase (Huang et al., 2017), prephenate aminotransferase (Pagnussat et al., 2005) and arogenate dehydratase (El-Azaz et al., 2018), have been shown to be essential for embryo development.

The biosynthesis of arginine occurs in plastids from glutamine and glutamate via a cyclic pathway leading to ornithine synthesis and a linear pathway converting ornithine to arginine. The whole pathway is regulated by allosteric inhibition of N-acetylglutamate kinase (NAGK) and ornithine transcarbamylase (OTC) by arginine (Llebrés et al., 2018b; Urbano-Gámez et al., 2020). However, when high levels of nitrogen (N) are available, the protein sensor PII interacts with NAGK to relieve this feedback control, increasing the metabolic flux through the pathway for N storage (Llaìcer et al., 2008). Two isoproteins of PII, PIIa, and PIIb, have been described in maritime pine, with PIIa being the predominant form in developing embryos (Llebrés et al., 2020). Compared to zygotic embryos, the enhanced expression of *ASS* and *ARG* genes in somatic embryos (Figure 7) is consistent with the simultaneous synthesis and degradation of arginine, as previously suggested (Pérez-Rodríguez et al., 2006; Llebrés et al., 2018b). However, other metabolic destinations for arginine such as nitric oxide, polyamines, or as precursor in proline metabolism, cannot be ruled out (Urbano-Gámez et al., 2020). Furthermore, the results also correspond to the increased ammonium reassimilation by GS1b for glutamine and asparagine biosynthesis (Cánovas et al., 2007). In fact, the release of urea and ornithine through the action of ARG and subsequent catabolism, in coordination with the activity of P5CDH, could provide not only ammonium but also glutamate for GS1b activity (Cañas et al., 2008). Asparagine is also important in N storage and mobilization in pine (Canales et al., 2012), and the observed decrease in the abundance of *ASPG* transcripts is consistent with the homeostasis of asparagine (Van Kerckhoven et al., 2017; Figure 7).

Another source of ammonium to be reassimilated by GS1b would be the deamination of phenylalanine for the biosynthesis of phenylpropanoids (Craven-Bartle et al., 2013; Pascual et al., 2016), a metabolic pathway highly induced in somatic embryos. The downregulation of *ADH* in the embryogenic lines suggests that tyrosine biosynthesis is not operative, and therefore, most of the arogenate from the shikimate pathway is channeled to phenylalanine biosynthesis. The existence of a more complex secondary metabolism in conifers (Warren et al., 2015) with more genes involved in phenylalanine utilization has been suggested to be correlated with a more complex family of ADT/PDT enzymes in plants (El-Azaz et al., 2016). In fact, the *ADT* gene family is in pine represented by more members than in angiosperms (El-Azaz et al., 2016, 2020). *ADT-F* and particularly *ADT-G* were downregulated in all the embryogenic lines (Figure 8). *ADT-F* is a conifer-specific gene of unknown function; however, *ADT-G* is an ortholog of *ADT2*, an essential gene for seed development in *Arabidopsis* (El-Azaz et al., 2018). As *ADT-G* encodes a bifunctional enzyme exhibiting ADT and PDT activities (El-Azaz et al., 2016), these results suggest that the phenylpyruvate pathway would be blocked, and therefore, the biosynthesis of phenylalanine would take place preferentially through the arogenate pathway. While ADT-G is strongly regulated by feedback control of its reaction product, other members of the ADT family are deregulated and therefore able to produce massive levels of phenylalanine which are channeled for increased phenylpropanoid biosynthesis (El-Azaz et al., 2020, 2021). The induction of *PAL*, *4CL*, *CSE*, and *CAD* genes in the embryogenic lines is consistent with the above hypothesis. This induction can be considered a response to changes in the abiotic environment that is likely mediated by increased biosynthesis of diverse secondary metabolites, as previously reported in woody plants (Berini et al., 2018), including during conifer embryogenesis [reviewed in Trontin et al. (2016c)]. Compared to the ZE embedded in the maternal megagametophyte which supplies nutrients and growth regulators in a dynamic way, the spatio-temporal culture environment of somatic embryos appears to be much more static and subjected to abiotic stress effects, in particular oxidative stress.

In summary, global analysis of transcriptome dynamics revealed striking differences in gene expression during late embryogenesis of somatic and zygotic embryos. Differentially regulated genes were clustered in different gene modules, and gene regulatory networks were identified and validated. Several embryogenic lines displayed changes in the regulation of metabolic pathways involved in amino acid biosynthesis, suggesting a reprogramming of N metabolism during SE in maritime pine.

## Data Availability Statement

The original contributions presented in the study are publicly available. This data can be found here: https://www.ncbi.nlm.nih.gov/geo/query/acc.cgi?acc=GSE194039.

## Author Contributions

ML and VC-R performed the experimental work at Universidad de Málaga and prepared the figures. IR and J-FT performed the experimental work at FCBA. CL-F performed the bioinformatic analysis of data. CÁ, FC, and J-FT conceived the project. CÁ and FC supervised the work and wrote the manuscript with contributions from J-FT, LH, ML, and VC-R. All authors contributed to the article and approved the submitted version.

## Conflict of Interest

The authors declare that the research was conducted in the absence of any commercial or financial relationships that could be construed as a potential conflict of interest.

## Publisher’s Note

All claims expressed in this article are solely those of the authors and do not necessarily represent those of their affiliated organizations, or those of the publisher, the editors and the reviewers. Any product that may be evaluated in this article, or claim that may be made by its manufacturer, is not guaranteed or endorsed by the publisher.

## Footnotes

1
https://github.com/rafnunser/seqtrimbb

2
https://github.com/vsbuffalo/sam2counts

3
https://www.ncbi.nlm.nih.gov/geo/query/acc.cgi?acc=GSE194039

4
https://github.com/ceslobfer/PinasterExpressionApp

## Supplementary Material

Supplementary Figure 1 Validation of differentially expressed transcripts by qPCR.

Supplementary Figure 2 Pathway map of nitrogen and arginine metabolism. A general overview of the metabolic reactions involved is shown. Differentially regulated steps are colored in the map.

Supplementary Figure 3 Pathway map of phenylpropanoid metabolism. A general overview of the metabolic reactions involved is shown. Differentially regulated steps are colored in the map.

Supplementary Table 1 Samples of somatic and zygotic embryos used for RNA sequencing.

Supplementary Table 2 List of the primer sequences used for transcript quantitative analysis.

Supplementary Table 3 List of hub genes.

Supplementary Table 4 List of hub TFs.

Supplementary File 1 Gene distribution in co-expression modules.

Supplementary File 2 Distribution of functional categories in gene modules.

Supplementary File 3 Genes differentially expressed in zygotic embryos.

Supplementary File 4 Genes differentially expressed in somatic embryos.
