# Supplementary figures and images for: Identification of Metabolic Pathways Differentially Regulated in Somatic and Zygotic Embryos of Maritime Pine
Source: Front Plant Sci. 2022 May 18;13:877960. doi: 10.3389/fpls.2022.877960 (PMC9159154; doi:10.3389/fpls.2022.877960)

## Slide 1
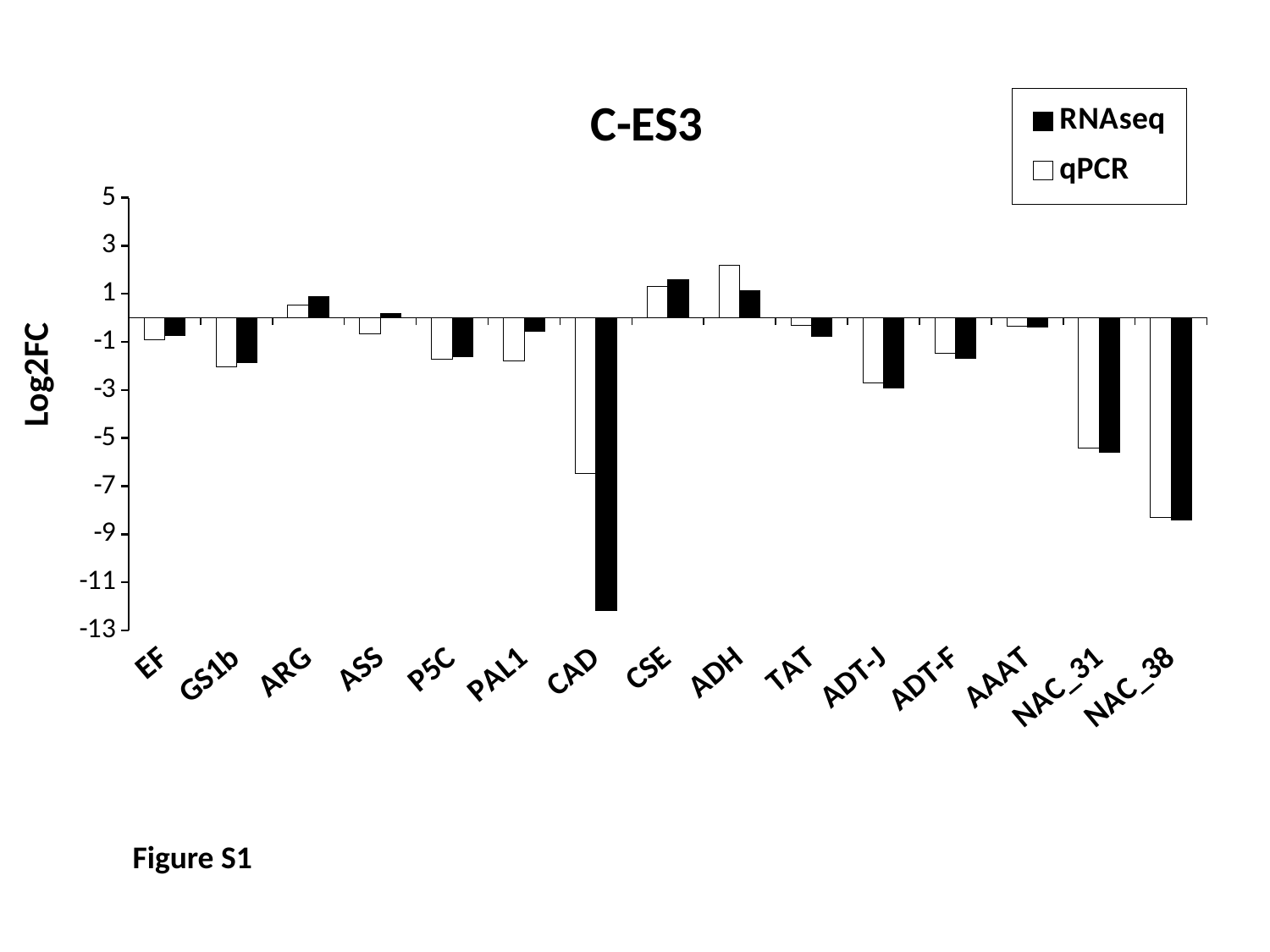

### Chart: C-ES3
| Category | | |
|---|---|---|
| NAC_38 | -8.4 | -8.3 |
| NAC_31 | -5.6 | -5.43 |
| AAAT | -0.38 | -0.364 |
| ADT-F | -1.69 | -1.49 |
| ADT-J | -2.91 | -2.71 |
| TAT | -0.76 | -0.3 |
| ADH | 1.12 | 2.2 |
| CSE | 1.57 | 1.29 |
| CAD | -12.16 | -6.47 |
| PAL1 | -0.57 | -1.79 |
| P5C | -1.6 | -1.72 |
| ASS | 0.18 | -0.65 |
| ARG | 0.89 | 0.549 |
| GS1b | -1.86 | -2.05 |
| EF | -0.75 | -0.93 |Figure S1

Supplement: Supplementary Figure 1 — Validation of differentially expressed transcripts by qPCR. [file Presentation_1.pptx]

## Slide 1
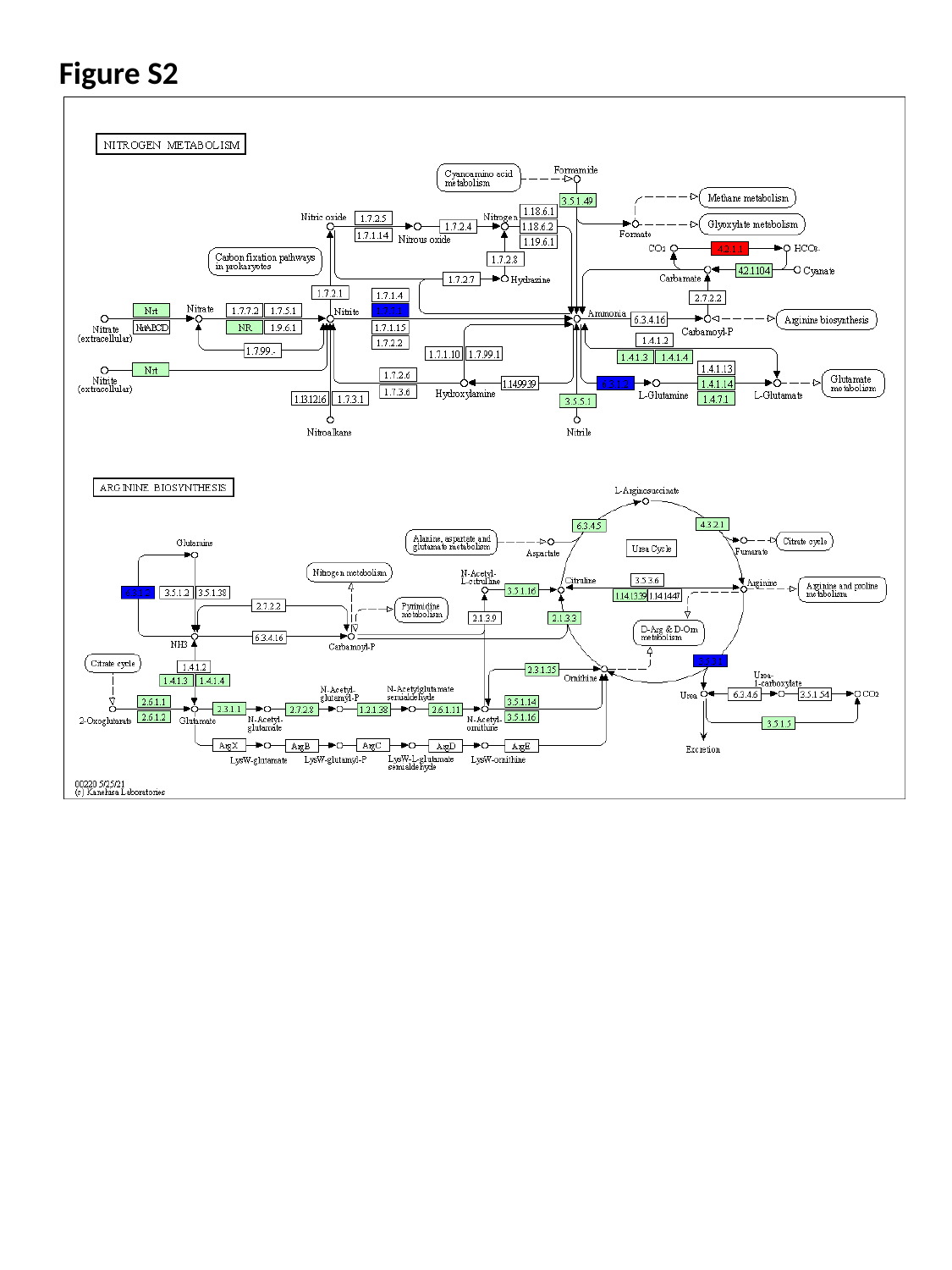

Figure S2

Supplement: Supplementary Figure 2 — Pathway map of nitrogen and arginine metabolism. A general overview of the metabolic reactions involved is shown. Differentially regulated steps are colored in the map. [file Presentation_2.pptx]

## Slide 1
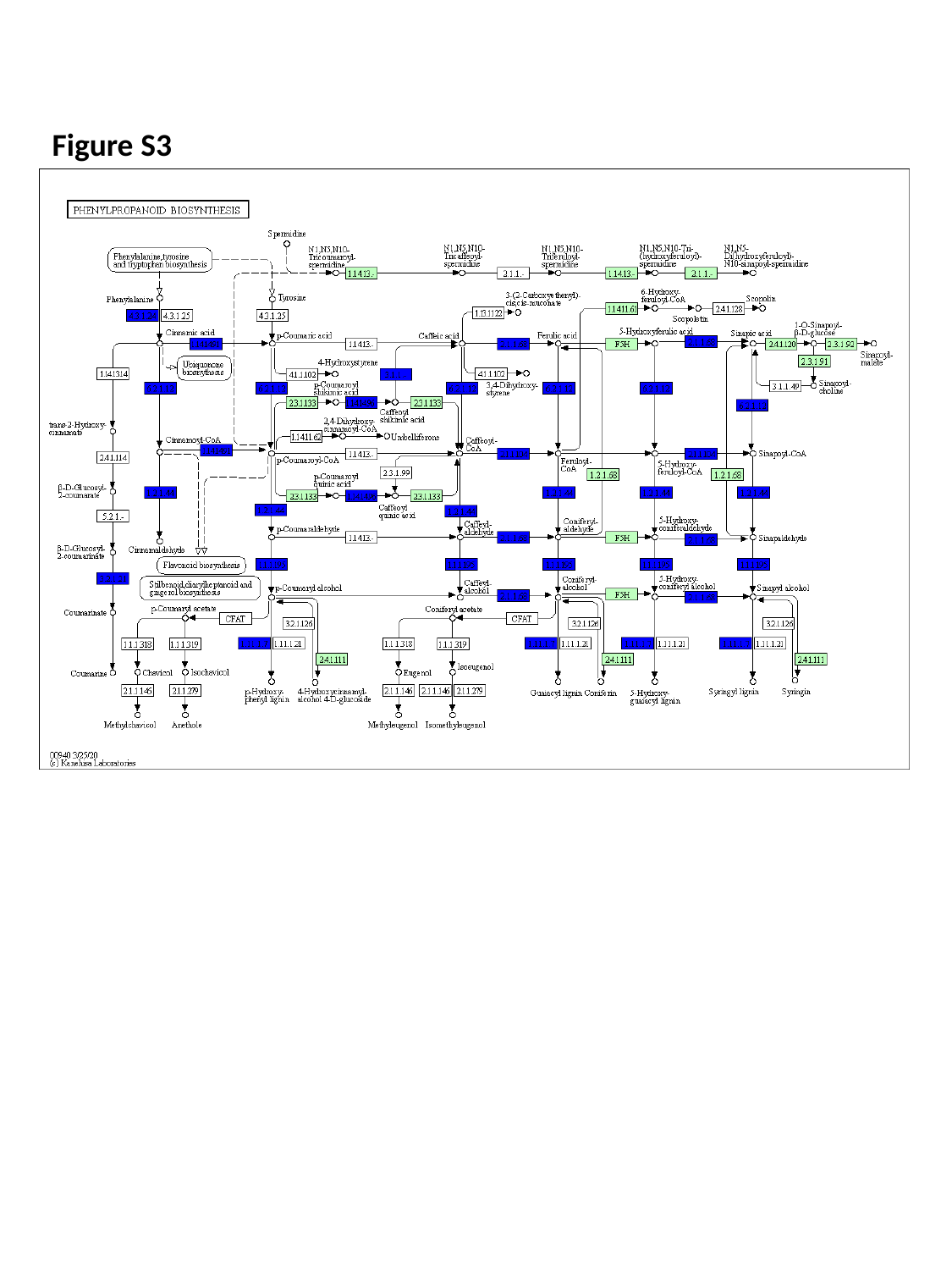

Figure S3

Supplement: Supplementary Figure 3 — Pathway map of phenylpropanoid metabolism. A general overview of the metabolic reactions involved is shown. Differentially regulated steps are colored in the map. [file Presentation_3.pptx]
