# Supplementary material for: Identification of Metabolic Pathways Differentially Regulated in Somatic and Zygotic Embryos of Maritime Pine
Source: Front Plant Sci. 2022 May 18;13:877960. doi: 10.3389/fpls.2022.877960 (PMC9159154; doi:10.3389/fpls.2022.877960)
Supplement: Supplementary Table 1 — Samples of somatic and zygotic embryos used for RNA sequencing. [file Table_1.docx]

Supplementary Table 1. Samples of somatic and zygotic embryos used for RNA sequencing

| **Name** | **Description** | **Organism** | **Provenance** |
| --- | --- | --- | --- |
| **ES1A** | Early-stage translucent somatic embryos collected after 4-6 weeks of maturation | *Pinus pinaster* | Somatic line PN519 |
| **ES1B** | Early-stage translucent somatic embryos collected after 4-6 weeks of maturation | *Pinus pinaster* | Somatic line PN519 |
| **ES1C** | Early-stage translucent somatic embryos collected after 4-6 weeks of maturation | *Pinus pinaster* | Somatic line PN519 |
| **ES2A** | Pre-cotyledonary opaque somatic embryos collected after 6-10 weeks of maturation | *Pinus pinaster* | Somatic line PN519 |
| **ES2B** | Pre-cotyledonary opaque somatic embryos collected after 6-10 weeks of maturation | *Pinus pinaster* | Somatic line PN519 |
| **ES2C** | Pre-cotyledonary opaque somatic embryos collected after 6-10 weeks of maturation | *Pinus pinaster* | Somatic line PN519 |
| **ES3A** | Cotyledonary somatic embryos collected after 12-14 weeks of maturation | *Pinus pinaster* | Somatic line PN519 |
| **ES3B** | Cotyledonary somatic embryos collected after 12-14 weeks of maturation | *Pinus pinaster* | Somatic line PN519 |
| **ES3C** | Cotyledonary somatic embryos collected after 12-14 weeks of maturation | *Pinus pinaster* | Somatic line PN519 |
| **PC1** | Pre-cotyledonary zygotic embryos collected early to mid-July | *Pinus pinaster* | Seeds collected from a single seed orchard from July to November 2015 |
| **PC2** | Pre-cotyledonary zygotic embryos collected early to mid-July | *Pinus pinaster* | Seeds collected from a single seed orchard from July to November 2015 |
| **PC3** | Pre-cotyledonary zygotic embryos collected early to mid-July | *Pinus pinaster* | Seeds collected from a single seed orchard from July to November 2015 |
| **EC1** | Early-cotyledonary zygotic embryos collected mid to late-July | *Pinus pinaster* | Seeds collected from a single seed orchard from July to November 2015 |
| **EC2** | Early-cotyledonary zygotic embryos collected mid to late-July | *Pinus pinaster* | Seeds collected from a single seed orchard from July to November 2015 |
| **EC3** | Early-cotyledonary zygotic embryos collected mid to late-July | *Pinus pinaster* | Seeds collected from a single seed orchard from July to November 2015 |
| **C1** | Cotyledonary zygotic embryos collected from early August to early September | *Pinus pinaster* | Seeds collected from a single seed orchard from July to November 2015 |
| **C2** | Cotyledonary zygotic embryos collected from early August to early September | *Pinus pinaster* | Seeds collected from a single seed orchard from July to November 2015 |
| **C3** | Cotyledonary zygotic embryos collected from early August to early September | *Pinus pinaster* | Seeds collected from a single seed orchard from July to November 2015 |
