# Supplementary material for: Identification of Metabolic Pathways Differentially Regulated in Somatic and Zygotic Embryos of Maritime Pine
Source: Front Plant Sci. 2022 May 18;13:877960. doi: 10.3389/fpls.2022.877960 (PMC9159154; doi:10.3389/fpls.2022.877960)
Supplement: Supplementary Table 2 — List of the primer sequences used for transcript quantitative analysis. [file Table_2.docx]

Supplementary Table 2. List of the primer sequences used for transcript quantitative analysis

| **Name** | **Description** | **Fwd-Primer** | **Rev-Primer** |
| --- | --- | --- | --- |
| ***Actin-7*** | *Actin-7* | ATCTCTCAGCACATTCCAACAG | TATTGCCACCATCATCTCAAGC |
| ***GS1b*** | *Glutamine synthetase b* | CAGCTTCCAACATGGACCCCTAT | TGGGGATCCCTTCGCAATTTGAC |
| ***P5CDH*** | *Pyrroline-5-carboxylate dehydrogenase* | TGTGAACGAATGCATGCCCA | GGAGCTCCTGTTGTTCGTGCA |
| ***ASPG*** | *Asparaginase* | GGAAGATGGTAAGGGTGGCC | AGGCTCTGAACATTCCCGAA |
| ***ASS*** | *Argininosuccinate synthetase* | TCCAATCAGAGTTCGAGCCA | GCTGAACTGATGCCTTATTGAC |
| ***ARG*** | *Arginase* | CGCGACACAGTTGATGGAAT | TGTCGAACTTCCTCCTGTTACA |
| ***PAL*** | *Phenylalanine ammonia-lyase* | TCGAATCTAAGTGGCGGGCCTAAT | TACATGGGTGGTGACAGGATTTGC |
| ***ADH*** | *Arogenate dehydratase* | TGTTGGTTGACTATTGTGGCCA | TGTTCTGCCGTCCCTTGGGA |
| ***CAD*** | *Cynnamyl-alcohol dehydrogenase* | TGCATGGTTGAGATTGTGGGTC | GTCCACGACGAACCTGTAACGA |
| ***CSE*** | *Caffeoyl shikimate esterase* | CATGGTTGGGCGAGAGAAGT | CCGACATCATGGCCGATTCA |
| ***4CL*** | *4-coumarate: CoA ligase 1* | CGCAATTCCCAAGTCTCCGGCT | TCAACGGTGGAGGTGGAGGT |
| ***ADT-G*** | *Arogenate dehydratase G isoform* | GTTCATGTAACTGAGAACTGGG | AACTTAGTGTCACACCCTCTC |
| ***ADT-F*** | *Arogenate dehydratase F isoform* | CTCCGTTCTTGTGACAGATCG | TTTGATCCTCAAATCCCTGTAC |
| ***ADT-J*** | *Arogenate dehydratase J isoform* | AGCACATTGTTTTCCACTGCCCT | CCAACTGAGCACTGCGGGAA |
| ***EF*** | *Elongation factor* | TGCTGTTGGAGTCATCAAGG | CATTTACCCTTCTTGGCCGC |
| ***TAT*** | *Tyrosine aminotransferase* | CAGGCTGCAATTCCACAAATCA | GCTGCACGGCTCAATAATTGC |
| ***AAAT*** | *Amino acid aminotransferase* | AGCATGGGATGGACAATGGAGGT | TGTAGGTCTGAGTGCCACTGA |
| ***NAC_38*** | *Transcription factor NAC_38* | CCAAGGAAATCAGATACGTGG | CTGATGTTGTATGATCTAGTCC |
| ***NAC_31*** | *Transcription factor NAC_31* | GATATGTCATCTGTAGTTTCG | GAAACAGCACAGTGAATTGGT |
