# Supplementary material for: Identification of Metabolic Pathways Differentially Regulated in Somatic and Zygotic Embryos of Maritime Pine
Source: Front Plant Sci. 2022 May 18;13:877960. doi: 10.3389/fpls.2022.877960 (PMC9159154; doi:10.3389/fpls.2022.877960)
Supplement: Supplementary Table 4 — List of hub TFs. [file Table_4.docx]

Supplementary Table 4. List of hub TFs

| **Module** | **Gene ID** | **TF family** | **Annotation** |
| --- | --- | --- | --- |
| BLACK | ppt_207949 | NAC | NAC domain protein (Pinus massoniana) |
| BLACK | ppt_207946 | NAC | NAC domain protein (Pinus massoniana) |
| BLUE | ppt_129368 | MYB | Transcription factor MYB31 (Norway spruce) |
| BLUE | ppt_129364 | MYB | Transcription factor TT2 (Morus notabilis) |
| BLUE | ppt_129365 | MYB | Transcription factor TT2 (Morus notabilis) |
| BLUE | ppt_129370 | MYB | Transcription factor TT2 (Morus notabilis) |
| BLUE | ppt_226590 | H2A | Histone H2A Glycine soja (Wild soybean) |
| BLUE | ppt_231182 |  | Histone H3.3 Vitis vinifera (Grape) |
| BLUE | ppt_90770 |  | Histone H2A.1 Solanum lycopersicum (Tomato) |
| BLUE | ppt_132360 |  | Uncharacterized protein (Hoop pine) |
| BLUE | ppt_47242 | ERF | Ethylene-responsive transcription factor ERF012  (Arabidopsis thaliana) |
| BLUE | ppt_231180 | H3.3 | Histone H3.3 Vitis vinifera (Grape) |
| BLUE | ppt_169677 | MYB | Myb-related protein Myb4 (Oryza sativa ) |
| BLUE | ppt_46902 |  | GATA transcription factor (Barbados nut) |
| BLUE | ppt_90101 | ERF | Putative uncharacterized protein (Picea sitchensis) |
| BLUE | ppt_90102 | ERF | Putative uncharacterized protein (Picea sitchensis) |
| BLUE | ppt_42141 | NAC | NAC transcription factor 32 (Arabidopsis thaliana) |
| BLUE | ppt_202456 | bHLH | Transcription factor bHLH62 (Pigeon pea) |
| BLUE | ppt_101962 | MYB | Myb-related protein Myb4 (Oryza sativa) |
| BLUE | ppt_155250 | ERF | Putative uncharacterized protein (Picea sitchensis) |
| BLUE | ppt_176960 | ERF | Putative uncharacterized protein (Picea sitchensis) |
| BLUE | ppt_202457 | bHLH | Transcription factor bHLH62 (Pigeon pea) |
| BLUE | ppt_183181 | NAC | NAC domain-containing protein 100 (Arabidopsis thaliana) |
| BLUE | ppt_183182 |  | Protein CUP-SHAPED COTYLEDON 2 (Arabidopsis thaliana) |
| BLUE | ppt_176959 | ERF | Putative uncharacterized protein (Picea sitchensis) |
| BLUE | ppt_117302 |  | Putative uncharacterized protein (Picea sitchensis) |
| BLUE | ppt_42454 |  | Putative uncharacterized protein (Picea sitchensis) |
| BLUE | ppt_3095 | ERF | Ethylene-responsive transcription factor ERF012  (Arabidopsis thaliana) |
| BLUE | ppt_72461 | b-Zip | LINE-1 reverse transcriptase isogeny (Pigeon pea) |
| BLUE | ppt_37549 |  | Putative uncharacterized protein (Vitis vinifera) |
| BLUE | ppt_148010 | ERF | Putative uncharacterized protein (Picea sitchensis) |
| BLUE | ppt_72463 | b-Zip | LINE-1 reverse transcriptase isogeny (Cajanus cajan) |
| BLUE | ppt_155247 | ERF | Putative uncharacterized protein (Picea sitchensis) |
| BLUE | ppt_171237 | ERF | ERF-like (Tarenaya spinosa) |
| BLUE | ppt_37547 |  | Putative uncharacterized protein (Vitis vinifera) |
| BLUE | ppt_158132 | H3.3 | Histone H3.3 (Glycine soja) |
| BROWN | ppt_241345 | MYB | Transcription factor MYB30 (Norway spruce) |
| BROWN | ppt_192238 | BEARSKIN | Protein BEARSKIN1 (Glycine soja) |
| GREEN | ppt_230051 | HMG | High mobility group B protein 7 (Glycine soja) |
| GREEN | ppt_230052 | HMG | High mobility group B protein 7 (Glycine soja) |
| GREEN | ppt_230049 | HMG | High mobility group B protein 7 (Glycine soja) |
| GREEN | ppt_230053 | HMG | High mobility group B protein 7 (Anthurium amnícola) |
| GREEN | ppt_230055 | HMG | High mobility group B protein 7 (Anthurium amnícola) |
| GREEN | ppt_230048 | HMG | High mobility group B protein 7 (Anthurium amnícola) |
| GREEN | ppt_230054 | HMG | High mobility group B protein 7 (Anthurium amnícola) |
| GREEN | ppt_97762 | DNApol | DNA polymerase delta small subunit (Arabidopsis thaliana) |
| GREEN | ppt_224322 | CDT1 | CDT1 A, putative (Theobroma cacao) |
| GREEN | ppt_97767 | DNApol | DNA polymerase delta small subunit (Arabidopsis thaliana) |
| GREEN | ppt_224321 | CDT1 | CDT1 A, putative (Theobroma cacao) |
| GREEN | ppt_72236 | DNApol | DNA polymerase delta catalytic subunit (Oryza sativa) |
| GREEN | ppt_152102 | UPF1 | Regulator of nonsense transcripts 1-like protein Chrysochromulina sp. CCMP291 |
| MAGENTA | ppt_58155 | NAC | NAC protein 1 splice variant 1 (Oil palm) |
| MAGENTA | ppt_58156 |  | Putative uncharacterized protein (Picea sitchensis) |
| MAGENTA | ppt_58154 |  | Putative uncharacterized protein (Picea sitchensis) |
| TURQUOISE | ppt_126099 |  | Wollemia nobilis Ref_Wollemi_Transcript_11459_2238 transcribed RNA sequence |
| TURQUOISE | ppt_126091 | B-ARR | Uncharacterized protein (Musa malaccensis) |
| TURQUOISE | ppt_126093 | B-ARR | Uncharacterized protein (Musa malaccensis) |
| TURQUOISE | ppt_182955 | AT-hook | AT-hook motif nuclear-localized protein 20 (Arabidopsis thaliana) |
| TURQUOISE | ppt_183013 | MYB | R2R3-MYB transcription factor MYB5 (Picea glauca) |
| TURQUOISE | ppt_182952 | AT-hook | AT-hook motif nuclear-localized protein 20 (Arabidopsis thaliana) |
| TURQUOISE | ppt_182956 | AT-hook | AT-hook motif nuclear-localized protein 20 (Arabidopsis thaliana) |
| TURQUOISE | ppt_175998 | C2H2 | Wollemia nobilis Ref_Wollemi_Transcript_12026_2455 transcribed RNA sequence Wollemia nobilis |
| YELLOW | ppt_123397 |  | Wollemia nobilis Ref_Wollemi_Transcript_25871_1634 transcribed RNA sequence Wollemia nobilis |
